# A spontaneous *TIR1* loss-of-function allele in *C. elegans*

**DOI:** 10.17912/micropub.biology.000994

**Published:** 2023-10-15

**Authors:** Krista M. Myles, An A. Vo, James Matthew Ragle, Jordan D. Ward

**Affiliations:** 1 Department of Molecular, Cell, and Developmental Biology, University of California, Santa Cruz, Santa Cruz, California, United States

## Abstract

The auxin-inducible degron (AID) system is a widely-used system for conditional protein depletion. During the course of an experiment, we depleted the nuclear hormone receptor transcription factor NHR-23 to study molting, and we recovered a spontaneous suppressor allele that bypassed the L1 larval arrest caused by NHR-23 depletion. These mutants also failed to deplete a BFP::AID reporter in the strain background, suggesting a broader defect in the AID system. These animals carried an in-frame 18 base pair insertion that produced a 6 amino acid repeat in TIR1. The larval arrest in these animals could be restored by expressing a wild-type *TIR1* transgene from an extrachromosomal array. Sister siblings that lost this array developed normally on auxin. Together, these experiments indicate that the *TIR1* mutation was causing the loss of developmental arrest in the *nhr-23::AID* strain. This result highlights the importance of setting up a robust secondary screen to detect such mutants if performing forward genetic screens in conjunction with the AID system.

**Figure 1. A TIR1 mutant allele causes loss of larval arrest in an nhr-23::AID, TIR1 strain.**
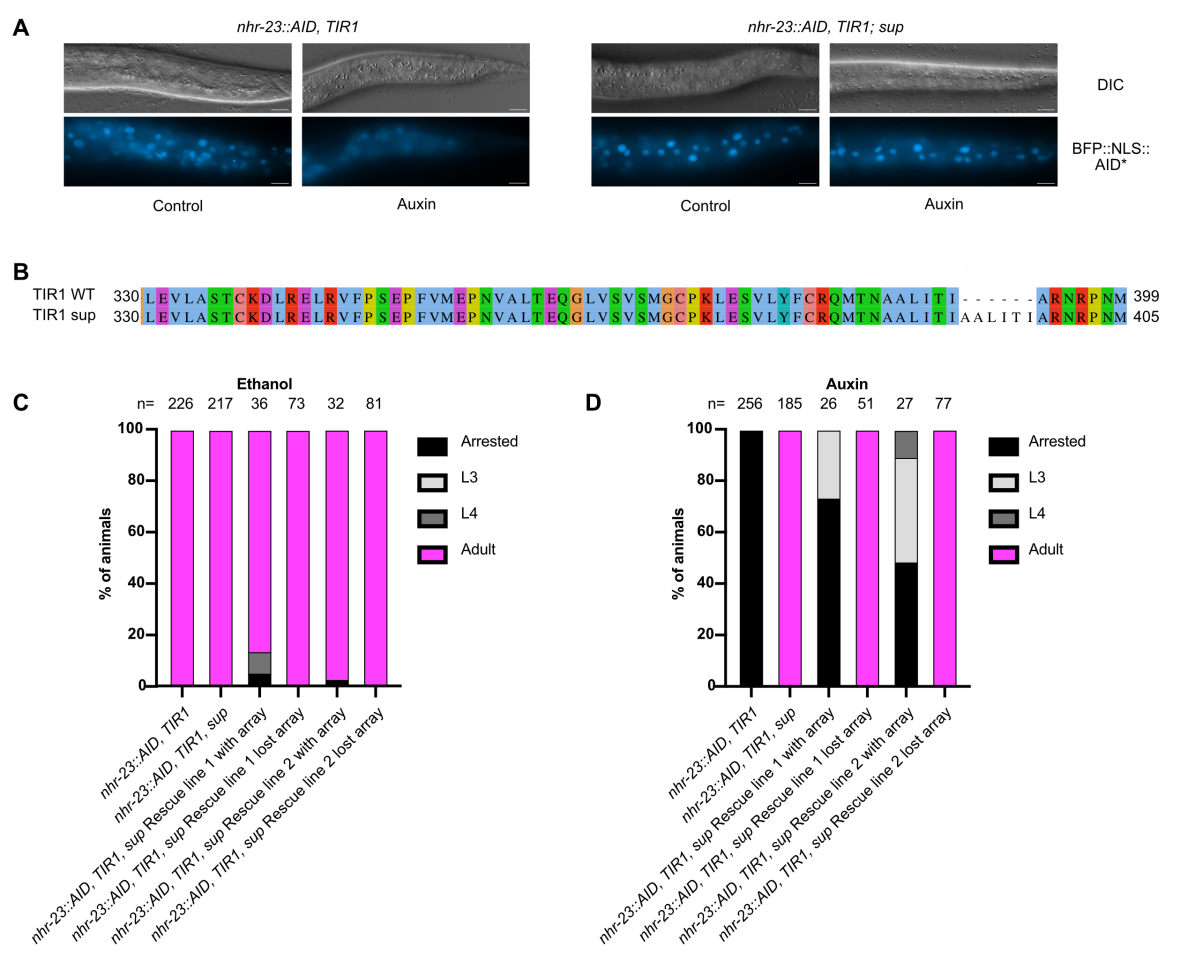
A) DIC and BFP images of the indicated genotypes grown on control or auxin plates. Scale bars=10 µm. (B) Amino acid sequence alignment of wild-type (WT) and candidate suppressor allele TIR (sup). Numbers indicate amino acid position within protein. Alignments were performed using ClustalW and JalView (Sievers et al., 2011; Waterhouse et al., 2009). Clustal amino acid default coloring was used (blue=hydrophobic; red=positive charge; magenta=negative charge; green=polar; pink=cysteine; orange=glycine; cyan=aromatic; unconserved=white). For the developmental timing assay in C and D, synchronized animals of the indicated genotype were generated by a timed egg lay on control ethanol (C) or auxin (D) plates and developmental stage was scored 24 hours later. For the rescue lines, animals carrying the array and those that lost the array were distinguished by *mlc-1* promoter reporter activity and these populations were scored separately. The number of animals scored over three independent experiments is provided at the top of each column. We note that Rescue lines carrying the array have a smaller sample size due to low transmittance of the array.

## Description

The auxin-inducible degron (AID) system is a widely-used, powerful system for conditional protein depletion in a broad range of organisms and cell types (Brown et al., 2017; Camlin & Evans, 2019; Chen et al., 2018; Daniel et al., 2018; Holland et al., 2012; Natsume et al., 2016; Nishimura et al., 2009; Trost et al., 2016; Zhang et al., 2015) . It is comprised of a protein of interest tagged with a degron (AID) sequence from a plant transcriptional regulator and a transgene expressing a plant F box protein Transport Inhibitor Response 1 (TIR1) (Natsume & Kanemaki, 2017) . TIR1 can complex with an endogenous SCF ubiquitin ligase complex. In the presence of the plant hormone auxin, TIR1 will bind the degron and the SCF ligase will ubiquitinate the degron, leading to proteasome-mediated degradation of the protein of interest (Natsume & Kanemaki, 2017) . In multicellular organisms, expressing TIR1 with tissue-specific promoters allows conditional, tissue-specific degradation of AID-tagged proteins (Ashley et al., 2021; Zhang et al., 2015) . The AID system has continued to evolve with modifications addressing auxin-independent degradation or incomplete degradation of target proteins (Hills-Muckey et al., 2022; Li et al., 2019; Nishimura et al., 2020; Sathyan et al., 2019; Sepers et al., 2022; Xiao et al., 2023; Yesbolatova et al., 2020) .

The AID system has been widely adopted by the *C. elegans* community to study processes such as molting, developmental timing, organogenesis, and meiosis (Azzi et al., 2020; Joseph et al., 2020; Ragle et al., 2020, 2022; Zhang et al., 2018) . Further improvements to the system include water soluble auxins and modified auxins that can penetrate the eggshell (Martinez et al., 2020; Negishi et al., 2019) . We recently used this system to characterize the role of the nuclear hormone receptor transcription factor NHR-23 in apical extracellular matrix regeneration during molting. Depletion of NHR-23 caused very slow growth similar to RNAi knockdown of NHR-23 (Johnson et al., 2023; Macneil et al., 2013) . NHR-23 depleted animals eventually tried to molt and died, with the phenotype being essentially a larval arrest (Johnson et al., 2023) .

In our typical experiments, when synchronized L1s were released onto 4 mM auxin animals arrested as L1s with complete penetrance (Johnson et al., 2023) . However, during one experimental replicate using nhr-23::AID, TIR1::2A::BFP::NLS::AID*; nas-37p::GFP::PEST animals, we recovered viable adults after 3 days on auxin. We will refer to the parent strain as *nhr-23::AID, TIR* and the suppressor strain as *nhr-23::AID, TIR; sup* going forward. We confirmed the expected genotype of these animals, suggesting that we had recovered a spontaneous suppressor allele. Possible explanations for the insensitivity of these animals to auxin were mutation of: i) the *nhr-23* degron sequence; ii) *TIR1* or another component of the SCF ligase; or iii) a gene required for auxin import into cells. Our TIR1 strains produce a separate nuclear localized BFP::AID reporter from the same mRNA on which TIR is encoded, providing a readout of both TIR1 expression and activity (Ashley et al., 2021) . In the *nhr-23::AID,TIR* control animals we observed nuclear BFP expression when grown on control plates, and severely reduced expression upon auxin exposure ( Figure 1A ). In contrast, *nhr-23::AID, TIR; sup* animals exhibited robust nuclear BFP expression following growth on both control and auxin plates ( Figure 1A ). These data indicated that both NHR-23::AID and BFP::AID were not being depleted, suggesting a more global defect in the AID system. These data also suggested that *TIR1* was being expressed. We first sequenced the *TIR1* transgene and identified an in-frame 18 base pair insertion that would produce a 6 amino acid AALITI repeat between residues 392 and 393 in WT TIR1 ( Figure 1B ). Interestingly, this position is relatively near the location of the hypomorphic *tir1-2* mutant allele in *Arabidopsis* which causes a G441D substitution (Ruegger et al., 1998) .

To test whether this *TIR1* insertion was the suppressor allele, we injected animals with a *TIR1::mRuby* rescue transgene carrying a bright pan-muscle co-injection marker and recovered two lines (El Mouridi et al., 2020) . We performed timed egg lays on control and auxin plates to acquire synchronized animals and scored developmental stages three days later. We scored rescue animals carrying the array and their siblings that lost the array separately. On control plates all strains developed normally, though rescue line 1 animals carrying the array exhibited a low level of developmental delay ( Figure 1C ). *nhr-23::AID, TIR1* animals all arrested on auxin while the suppressor strain animals all reached adulthood, as expected ( Figure 1D ). Rescue animals with the array arrested or exhibited developmental delay and no animals made it to adulthood ( Figure 1D ). Strikingly, their siblings that lost the array all made it to adulthood similar to *nhr-23::AID, TIR1, sup* animals ( Figure 1D ). Together, these data strongly suggest that the *TIR1* insertion is the suppressor allele. While *nhr-23::AID, TIR1* animals exhibited a more penetrant arrest than the rescue lines carrying the array, this may be due to mosaicism. Our recovery of a spontaneous *TIR1* mutant in the absence of mutagenesis highlights the importance of building in secondary screens to detect such alleles should one perform forward genetic screens in conjunction with the AID system. It also highlights the value of our TIR1::2A::BFP::NLS::AID* strains for rapidly detecting mutations that globally affect the AID system.

## Methods

*C. elegans strains and culture*

*C. elegans* strains (see table in Reagents) were cultured as originally described (Brenner, 1974) , except worms were grown on MYOB instead of NGM. MYOB was made as previously described (Church et al., 1995) . For auxin depletion experiments, 0.25% ethanol or 4 mM auxin (indole-3-acetic acid; IAA; Alfa Aesar) were used. Plates were made as previously described (Johnson et al., 2023) . JDW445 animals were recovered by releasing JDW395 animals synchronized by alkaline bleaching ( dx.doi.org/10.17504/protocols.io.j8nlkkyxdl5r/v1 ) onto auxin plates. Three days later fertile adults were recovered. The presence of the *nas-37p::GFP::PEST* transgene was confirmed by fluorescence microscopy and the nhr-23::AID*::3xFLAG and *TIR1* alleles were confirmed by PCR genotyping with oligos 1586+1587+3380 and 2835+2836+3415 (see Reagents for sequences). A 64ºC annealing temperature and 45 second extension was used. Animals were cultured at 20°C for all assays, unless otherwise indicated. For general strain propagation, animals were grown at 15°C according to standard protocols.

*TIR1* rescue experiments were performed by amplifying *eft-3p::TIR1::mRuby::unc-54 3’UTR* from pLZ31 (Zhang et al., 2015) using oligos 7000+7001. The PCR product was purified using a Qiagen PCR clean up kit and injected into JDW445 at 5 ng/µl along with a pSEM228 co-injection marker (El Mouridi et al., 2020) . Marker positive animals were singled and two independent lines that propagated the extrachromosomal array were isolated. Developmental timing assays were performed by placing 2-8 adults of the indicated genotype per well in a 6-well ethanol or auxin plate for five hours at 20ºC. The parent animals were removed and plates were incubated for a further 72 hours at 20ºC before scoring for developmental stage.

*Microscopy*

Imaging was performed as previously described (Johnson et al., 2023) . Animals were synchronized by alkaline bleaching and released on control or auxin plates and incubated for 24 hours. Animals were picked into a 15 µl drop of M9+5 mM levamisole on a glass slide with a 2% agarose pad and secured with a coverslip. Animals were imaged using a Plan-Apochromat 63×/1.4 Oil DIC lens on an AxioImager M2 microscope (Carl Zeiss Microscopy) equipped with a Colibri 7 LED light source and an Axiocam 506 mono camera. We used Fiji software (version: 2.0.0- rc-69/1.52p) to process images (Schindelin et al., 2012) . For the comparisons in the developmental time course or between strains, we set the exposure conditions to avoid pixel saturation of the brightest sample and kept equivalent exposure for imaging of the other samples.

## Reagents

**Table:** 

**Strain**	**Genotype**	**Available from**
JDW395	wrdSi73[eft-3p::TIR1::F2A::mTagBFP2::AID*::NLS::tbb-2 3'UTR] ; nhr-23(kry61(nhr-23::AID*-TEV-3xFLAG)) I; oxIs134[Pnas-37::GFP:rODC(pest)(pWD95@90ng/ul),lin-15(+)]	Prof. Jordan Ward
JDW445	wrdSi73(wrd110[TiR1 aa391 AALITI insertion *wrdSi73]); nhr-23(kry61(nhr-23::AID*-TEV-3xFLAG)) I; oxIs134[Pnas-37::GFP:rODC(pest)(pWD95@90ng/ul),lin-15(+)]	Prof. Jordan Ward
JDW692	wrdSi73(wrd110[TiR1 aa391 AALITI insertion *wrdSi73]); nhr-23(kry61(nhr-23::AID*-TEV-3xFLAG)) I; oxIs134[Pnas-37::GFP:rODC(pest)(pWD95@90ng/ul),lin-15(+)]; wrdEx45[eft-3p::TIR1::mRuby2::unc-54 3'UTR + mlc-1p::mNeonGreen]	Prof. Jordan Ward
JDW693	wrdSi73(wrd110[TiR1 aa391 AALITI insertion *wrdSi73]); nhr-23(kry61(nhr-23::AID*-TEV-3xFLAG)) I; oxIs134[Pnas-37::GFP:rODC(pest)(pWD95@90ng/ul),lin-15(+)]; wrdEx45[eft-3p::TIR1::mRuby2::unc-54 3'UTR + mlc-1p::mNeonGreen]	Prof. Jordan Ward

**Table:** 

**Oligo number**	**Sequence (5' to 3')**	**Purpose**
1586	GTGTGCGGTGAAAGGTATTCTG	*nhr-23* knock-in genotyping
1587	AATGAGGAACTCTCCTGCAAC	*nhr-23* knock-in genotyping
2835	TGTCGACCGCTAGTGTAGCTTAC	TIR1 transgene genotyping
2836	CGTCTCTCCACGATTTACACACTATTTG	TIR1 transgene genotyping
3415	CTGGCCGTCGTTTTACAGGA	TIR1 transgene genotyping
3380	AAGAACGTGATGGTTTCCTGC	*nhr-23* knock-in genotyping
7000	TTCGCTGTCCTGTCACACTCG	Amplify *TIR1* rescue transgene
7001	TAAGGAGTTCCACGCCCAGG	Amplify *TIR1* rescue transgene
